# Ultrasound- versus Palpation-Guided Injection of Corticosteroid for Plantar Fasciitis: A Meta-Analysis

**DOI:** 10.1371/journal.pone.0092671

**Published:** 2014-03-21

**Authors:** Zonghuan Li, Chengyan Xia, Aixi Yu, Baiwen Qi

**Affiliations:** Department of Orthopedics, Zhongnan Hospital of Wuhan University, Wuhan, China; Iran University of Medical Sciences, Iran (Islamic Republic of)

## Abstract

**Background:**

It is controversial whether ultrasound-guided injection of corticosteroid is superior to palpation-guided injection for plantar fasciitis. This meta-analysis was performed to compare the effectiveness of ultrasound-guided and palpation-guided injection of corticosteroid for the treatment of plantar fasciitis.

**Methods:**

Databases (MEDLINE, Cochrane library and EMBASE) and reference lists were searched from their establishment to August 30, 2013 for randomized controlled trials (RCTs) comparing ultrasound-guided with palpation-guided injection for plantar fasciitis. The Cochrane risk of bias (ROB) tool was used to assess the methodological quality. Outcome measurements were visual analogue scale (VAS), tenderness threshold (TT), heel tenderness index (HTI), response rate, plantar fascia thickness (PFT), hypoechogenicity and heel pad thickness (HPT). The statistical analysis was performed with software RevMan 5.2 and Stata 12.0. When I^2^<50%, the fixed-effects model was adopted. Otherwise the randomized-effects model was adopted. The Grading of Recommendations Assessment, Development and Evaluation (GRADE) system was used to assess the quality of evidence.

**Results:**

Five RCTs with 149 patients were identified and analyzed. Compared with palpation-guided injection, ultrasound-guided injection was superior with regard to VAS, TT, response rate, PFT and hypoechogenicity. However, there was no statistical significance between the two groups for HPT and HTI.

**Conclusion:**

Ultrasound-guided injection of corticosteroid tends to be more effective than palpation-guided injection. However, it needs to be confirmed by further research.

## Introduction

Up to 10% adults will suffer heel pain during the lifetime[1] and about 80% patients are caused by plantar fasciitis.[2] Patients with plantar fasciitis feel heel pain when rising from bed and during initial weight-bearing in the morning. Generally, plantar fasciitis is treated conservatively with rest, nonsteroidal anti-inflammatory drug, stretching of plantar fascia, physical therapy, foot padding, et al.[3] After conservative treatment fails, injection of corticosteroid is a potential option. Palpation-guided injection is an effective and common treatment.[4] However, it is somewhat subjective and varies with the practitioners.^5^ It is not always successful and may need repeated injections occasionally, which may be accompanied with potential complications including fat pad atrophy and rupture of plantar fascia.[2] Real-time ultrasound is a noninvasive, relative low-cost method without radiation. The ultrasound image shows increased thickness and hypoechoic fascia, which is in accordance with pathological change of plantar fasciitis.[5] Ultrasound-guided injection provides a well-tolerated, dynamic and precise location of injection. Thus it is considered to be more effective than palpation-guided injection.[6], [7] However, several studies[8]-[10] reported that no difference in visual analogue score (VAS) following steroid injection between both groups.

Several published randomized controlled trials (RCTs) have compared ultrasound- and palpation-guided corticosteroid injection for the treatment of plantar fasciitis. However, the conclusions are inconsistent. The goal of this study is to perform a meta-analysis of the efficacy of ultrasound- and palpation guided injections.

## Methods

The study protocol was shown in Checklist S1.

### Search strategy

We systematically searched MEDLINE, Cochrane library and EMBASE for RCTs from their establishment to August 30, 2013. Studies comparing ultrasound-guided and palpation-guided corticosteroid injections for plantar fasciitis were selected. Medical Subject Headings together with the search terms (“plantar fasciitis” and “heel pain”, “painful heel”, “ultrasound”, “sonograph*”, “ultrasonography”, “palpation”, “unguided”, “blind”) were used (File S1). The reference lists were checked for additional studies.

Two reviewers (Li Z and Xia C) independently screened the titles and abstracts to identify potentially relevant studies. Full text of all identified studies were obtained and then reviewed. Studies met the eligibility criteria were selected. The final results were confirmed by two senior reviewers (Yu A and Qi B).

### Eligibility criteria

Eligibility criteria was established based on PICOS (patient, intervention, comparison, outcome and study design) as the following: (i) P: patients were diagnosed as plantar fasciitis based on heel pain and point tenderness over the medial tubercle of the calcaneus, which started with the first step in the morning, weakened thereafter, and worsened with weight-bearing activity; (ii) I and C: ultrasound-guided and palpation-guided injections were compared; (iii) O: one or more outcome(s) (VAS, tenderness threshold (TT), heel tenderness index (HTI), response rate, plantar fascial thickness (PFT), hypoechogenicity and heel pad thickness (HPT)) was (were) described; (iv) S: only RCTs were included. No language restriction was set.

The details of the outcomes were as follows. A VAS score was a 10-score or 100-score tool that tried to estimate the pain intensity. Zero represented no pain while 10 or 100 scores represented worst pain. TT was measured by a pressure algometer placed on the medial calcaneal tuberosity perpendicular to skin surface. The minimum pressure required to cause pain was defined as TT. If there was no pain at maximal pressure, the TT was 10 kg/cm^2^. HTI was an index that physician used to assess heel pain on palpation. It was defined as 0 = no pain, 1 = painful, 2 = painful and winces, and 3 = painful, winces and withdraws. The response rate referred to effective rate by one injection. PFT was measured with an electronic caliper from the proximal point of insertion of the fascia to the calcaneal tubercle. PFT of >4.5 mm was considered abnormal. Hypoechogenicity of the plantar fascia was recorded according to the ultrasound findings. HPT was measured by ultrasonography from the skin surface to the nearest calcaneal tuberosity.

### Data extraction

Data extraction was performed independently by two authors (Li Z and Xia C). The demographic characteristics (first author, published year, location, sample size, average age, male/female ratio, body mass index (BMI), intervention and study design) were extracted. All outcomes as mentioned above were extracted for meta-analysis.

### Methodological assessment

Two independent authors (Li Z and Xia C) evaluated the methodological quality of included studies with the risk of bias (ROB) tool provided by Cochrane collaboration[11]. The ROB tool consists of 7 items including random sequence generation, allocation concealment, blinding of participants and personnel, blinding of outcome assessment, incomplete outcome data, selective reporting and other bias. Each item was assigned a judgment of “low risk”, “unclear risk” and “high risk” based on the data provided by the article[11]. Namely, the judgment was “low risk” for the item with sufficient and correct information. And the judgment was “high risk” for the item reported incorrectly. If the information of the item was insufficient or unmentioned, the judgment was “unclear risk”. An “unclear risk” judgment should also be made if the item was reported, but the risk of bias is unknown. The disagreement was solved by a senior reviewer (Yu A).

### Statistical analysis

Outcomes were VAS, TT, HTI, response rate, PFT, hypoechogenicity and HPT. Statistical analysis was performed with software RevMan (version 5.2) and Stata (version 12.0) by two reviewers (Li Z and Qi B). Relative risk (RR) and mean difference (MD), both with 95% confidence intervals (CI), were adopted to analyze dichotomous data and continuous variables, respectively. The I^2^ value was used to estimate statistical heterogeneity. When I^2^<50%, heterogeneity could be accepted and the fixed-effects model was adopted. Otherwise the randomized-effects model was adopted. Publication bias was assessed by Egger's test. A P value <0.05 was considered statistically significant.

The quality of evidence was evaluated with GRADE system[12] (GRADEprofiler 3.6) by two reviewers (Li Z and Qi B). RCT was high-quality evidence. It could be downgraded for five reasons: risk of bias, inconsistency, indirectness, imprecision and publication bias. Finally, there were four levels of evidence quality, namely high, moderate, low and very low.

## Results

### Identification of relevant literature

**Figure 1:**
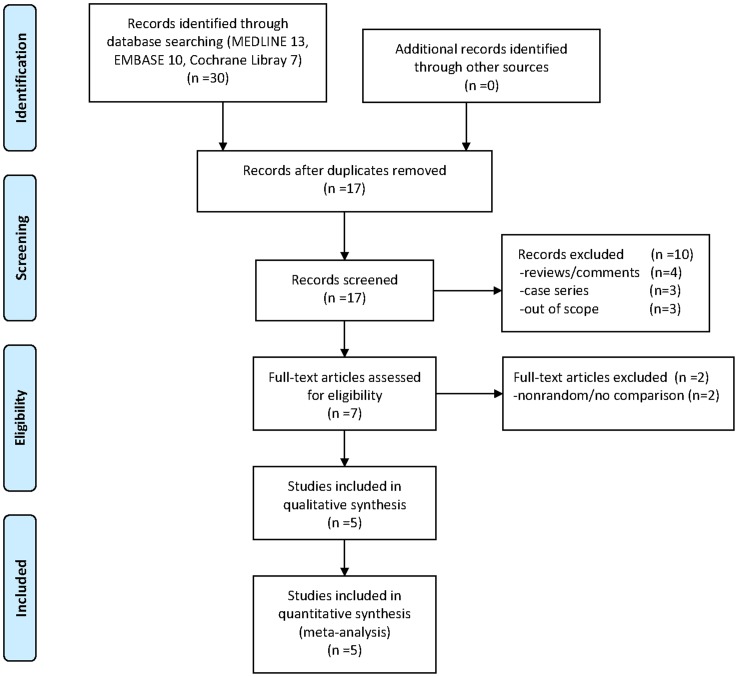
Flow diagram of the process of literature screening.

A total of 30 studies were retrieved from the database search and reference list check. Seventeen studies remained after the exclusion of 13 duplicate studies. Ten studies were excluded after examination by reviewing the title and abstract. Finally, after checking the full text, 5 RCTs[6]–[10] with 149 patients were included in this meta-analysis (Figure 1). Table 1 lists the general characteristics of all included studies. Most patients were middle aged, with an average age range from 46 to 58. The average BMI in all studies was greater than 25, which was considered overweight. All studies were RCTs and intervened with ultrasound-guided and palpation-guided injections for plantar fasciitis.

**Table 1:** General characteristics of included studies.

Included study	Location	Sample size	Mean age (years)	Mean BMI	Intervention	Comparison	Study design	Outcomes	Follow-up duration
Ball 2013	United Kingdom	44	49	30.7/31.8	ultrasound-guided injection of 0.5 ml (20 mg) methylprednisolone acetate and 0.5 ml saline	palpation-guided injection of 0.5 ml (20 mg) methylprednisolone acetate and 0.5 ml saline	RCT	VAS, HTI PFT, RR	12 weeks
Chen 2013	China	32	55	27.09/28.27	device-assisted ultrasound-guided injection of 7 mg (1 ml) betamethasone and 5 mg (0.5 ml) lidocaine	palpation-guided injection of 7 mg (1 ml) betamethasone and 5 mg (0.5 ml) lidocaine	RCT	VAS, TT, PFT, HPT, Hypoechogenicity	3 months
Kane 2001	Ireland	23	58	30.4	ultrasound-guided injection of triamcinolone hexacetonide and xylocaine into the plantar fascia	palpation-guided injection of triamcinolone hexacetonide and xylocaine into the plantar fascia	RCT	VAS, HTI, PFT, RR	13.4 weeks
Tsai 2006	China	25	51	25.1/26.9	sonographically-guided injection of 7 mg (1 ml) dexamethasone and 5 mg (0.5 ml) lidocaine	palpation-guided injection of 7 mg (1 ml) dexamethasone and 5 mg (0.5 ml) lidocaine	RCT	VAS, TT, PFT, RR, hypoechogenicity	1 year
Yucel 2009	Turkey	21	46	28.7/28.5	ultrasound-guided injection of 0.5 ml betamethasone dipropionate (6.43 mg/ml) and betamethasone sodium phosphate (2.63 mg/ml) combination, and 0.5ml prilocaine-HCl(20mg/ml)	palpation-guided injection of 0.5 ml betamethasone dipropionate (6.43 mg/ml) and betamethasone sodium phosphate (2.63 mg/ml) combination, and 0.5ml prilocaine-HCl(20mg/ml)	RCT	VAS, PFT, HPT, hypoechogenicity	25.3 months

−/−: ultrasound-guided injection/palpation-guided injection. BMI: body mass index; RCT: randomization controlled trial; VAS: visual analogue score; TT: tenderness threshold; HTI: heel tenderness index; PFT: plantar fascia thickness; HPT: heel pad thickness; RR: response rate.

### Methodological assessment

Study quality of included studies was showed in Table 2. Randomized sequence generation and single blind method were reported in one study. Other studies mentioned randomization, however, more detailed information was not available. Blind method was not used in one study. Incomplete outcome data and selective reporting were of low risk. The rest items were unclear.

**Table 2:** Risk of bias of included studies.

Included study	Random sequence generation	Allocation concealment	Blinding of patients	Blinding of therapists	Incomplete outcome data	Selective reporting	Other bias
Ball 2013	Low	Unclear	Low	Unclear	Low	Low	Unclear
Chen 2013	Unclear	Unclear	Unclear	High	Low	Low	Unclear
Kane 2001	Unclear	Unclear	Unclear	Unclear	Low	Low	Unclear
Tsai 2006	Unclear	Unclear	Unclear	Unclear	Low	Low	Unclear
Yucel 2009	Unclear	Unclear	Unclear	Unclear	Low	Low	Unclear

### Outcome measurements

**Figure 2:**
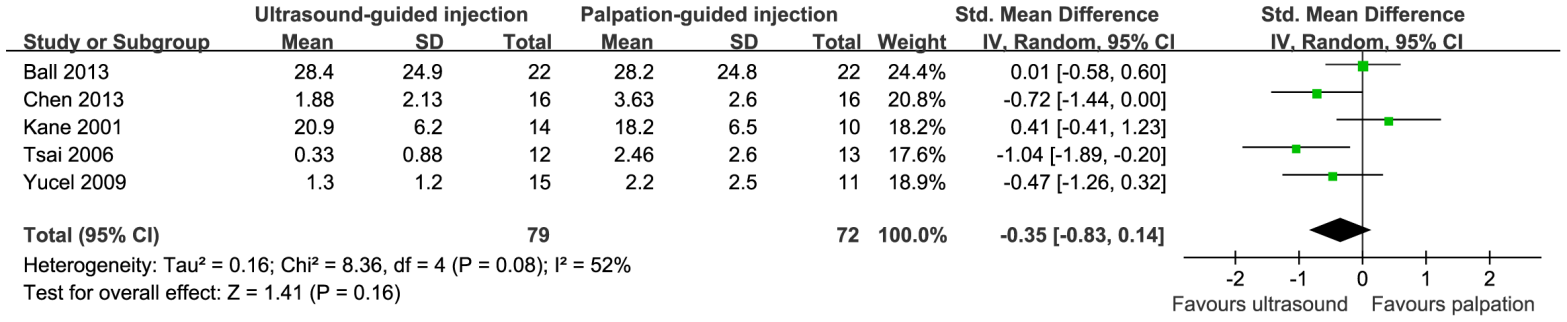
Forest plot for VAS score between ultrasound-guided injection and palpation-guided injection.

**Figure 3:**
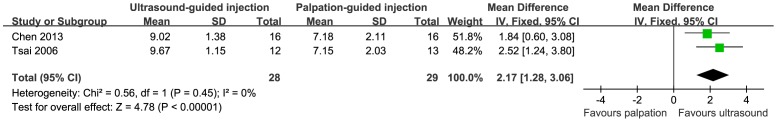
Forest plot for tenderness threshold between ultrasound-guided injection and palpation-guided injection.

**Figure 4:**
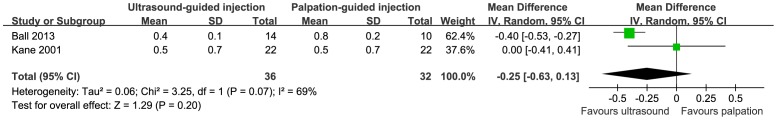
Forest plot for heel tenderness index between ultrasound-guided injection and palpation-guided injection.

**Figure 5:**
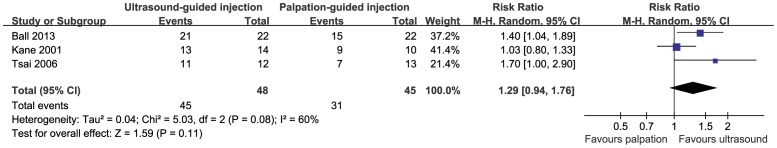
Forest plot for response rate between ultrasound-guided injection and palpation-guided injection.

VAS score, TT and HTI were reported in five, two and two studies, respectively. The results indicated that no difference was detected with respect to VAS score (SMD = −0.35, 95%CI (−0.83, 0.14), *P* = 0.16) (Figure 2). Compared with palpation-guided injection, ultrasound-guided injection showed improvement of pain symptom with higher TT (MD = 2.17, 95%CI (1.28, 3.06), *P = *0.00) (Figure 3). The difference of HTI in both groups did not achieve significantly (MD = −0.25, 95%CI (−0.63, 0.13), *P* = 0.20) (Figure 4). However, it showed a tendency of lower VAS score and lower HTI in ultrasound-guided injection group. Response rate was recorded in 3 studies with a total of 93 patients. Patients treated with ultrasound-guided injection showed a tendency of higher response rate though without significant difference (RR = 1.29, 95%CI (0.94, 1.76), *P* = 0.11) (Figure 5).

**Figure 6:**
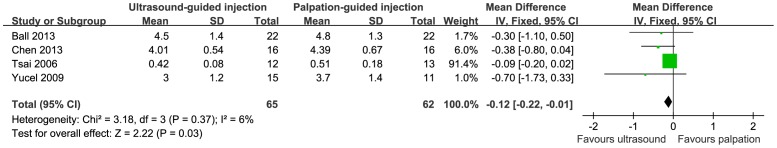
Forest plot for plantar fascia thickness between ultrasound-guided injection and palpation-guided injection.

**Figure 7:**
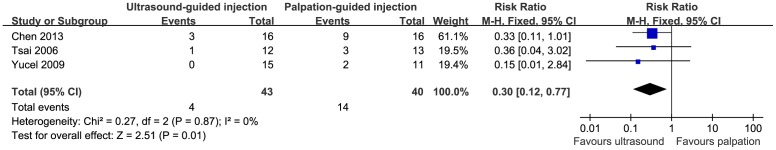
Forest plot for hypoechogenicity between ultrasound-guided injection and palpation-guided injection.

**Figure 8:**
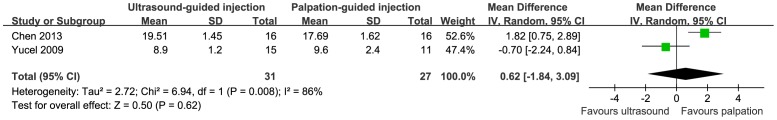
Forest plot for heel pad thickness between ultrasound-guided injection and palpation-guided injection.

Objective changes, including PFT, hypoechogenicity and HPT were recorded in four, three and two studies, respectively. All these outcomes were measured by ultrasound during the follow-up. PFT was thinner (MD = −0.12, 95%CI (−0.22, −0.01), *P* = 0.03) (Figure 6) and hypoechogenicity was less detected (RR = 0.30, 95%CI (0.12, 0.77), *P* = 0.01) (Figure 7) in ultrasound-guided injection group than those in palpation-guided injection group. However, HPT in both groups did not achieve significant difference (MD = 0.62, 95%CI (−1.84, 3.09), *P* = 0.62) (Figure 8), which meant no atrophy occurred.

### Publication bias

The Egger's test did not achieve significant difference with respect to all outcomes (Table S1). The results suggested that no publication bias existed.

### Quality of evidence

According to the GRADE system, the quality of evidence was moderate for VAS, TT, PFT and hypoechogenicity, low for response rate, HTI and HPT (Table 3).

**Table 3:** GRADE assessment of outcomes.

Outcome	Quality assessment							Quality	Importance
	No of studies	Design	Risk of bias	Inconsistency	Indirectness	Imprecision	Publication bias		
VAS	5	RCT	serious^a^	no serious inconsistency	no serious indirectness	no serious imprecision	Undetected	Moderate	Critical
TT	2	RCT	serious^a^	no serious inconsistency	no serious indirectness	no serious imprecision	Undetected	Moderate	Critical
Response rate	3	RCT	serious^a^	serious^b^	no serious indirectness	no serious imprecision	Undetected	Low	Critical
HTI	2	RCT	serious^a^	serious^b^	no serious indirectness	no serious imprecision	Undetected	Low	Important
PFT	4	RCT	serious^a^	no serious inconsistency	no serious indirectness	no serious imprecision	Undetected	Moderate	Important
HPT	2	RCT	serious^a^	serious^b^	no serious indirectness	no serious imprecision	Undetected	Low	Important
Hypoechogenicity	3	RCT	serious^a^	no serious inconsistency	no serious indirectness	no serious imprecision	Undetected	Moderate	Important

a Random sequence generation, allocation concealment and blind method were not reported in most studies.

b The heterogeneity among included studies was not neglectable.

RCT: randomization controlled trial; VAS: visual analogue score; TT: tenderness threshold; HTI: heel tenderness index; PFT: plantar fascia thickness; HPT: heel pad thickness.

## Discussion

Corticosteroid injection is an effective method for the management of plantar fasciitis.[13] However, it is still controversial whether ultrasound-guided injection is superior to palpation-guided injection. We thus identified five RCTs and conducted a meta-analysis. The results revealed that the patients in ultrasound-guided injection showed, higher TT, thinner PFT and lower incidence of hypoechogenicity. No obvious improvement occurred with respect to VAS score, HTI, HPT and response rate, though with an inclined favor for ultrasound-guided injection.

Generally, patients with plantar fasciitis are treated conservatively at their first presentation. After the conservative treatments fail, corticosteroid injection is potentially adopted. Palpation-guided injection has been confirmed as an effective and safe method. Genc H et al[4] treated 30 plantar fasciitis patients with palpation-guided injection. After the treatment, VAS score, PFT and the incidence of hypoechogenicity decreased significantly. However, complications including fascia rupture and heel pad atrophy, though uncommon, does exist. Magnetic resonance imaging provides clear morphological change of plantar fascia and distinguishes plantar fasciitis from heel pain by other causes.[14] It is still not suitable for serial follow-ups because of inconvenience and expensive cost. Bone scintigraphy is more specific but less sensitive than ultrasound for the diagnosis of plantar fasciitis.[6] However, scintigraphy-guided injection is not as efficient as ultrasound- and palpation-guided injections. Ultrasound is a relative quick, less expensive, no radiation exposure and widely available technique. It provides excellent delineation and real-time visualization for soft tissue. Normally, the ultrasound feature of plantar fasciitis is increased thickness, obscure borders and hypoechogenicity of plantar fascia.[5] Recently, real-time sonoelastography was applied to monitor plantar fasciitis and the diagnosis performance increased compared with B-model ultrasound.[15]

In this meta-analysis, patients with plantar fasciitis were diagnosed based on clinical manifestation. Age and obesity are risk factors for developing plantar fasciitis. The mean age of patients ranged from 46 to 58 and the mean BMI ranged from 25.1 to 31.8. It was in accordance with the ideal that most patients with plantar fasciitis were usually overweight and middle aged.[3] Plantar fasciitis is believed to be caused by repetitive microtrauma and degenerative changes in the plantar fascia are observed histologically.[16] Thus patients with age ranged from 40 to 60 are prone to suffered plantar fasciitis[3] and overweight accelerates the progress.

Several studies suggested that non-ultrasound-guided injection was cheaper and equally effective to ultrasound-guided injection.[13] The application of ultrasound for plantar fasciitis increased health care costs. Furthermore, the treatment for patients with plantar fasciitis was more complicated because the procedure needed both ultrasound specialist and clinician. Thus ultrasound-guided injection should be adjunct but not routine. However, results from other researches were in contradiction to them. Palpation-guided injection of steroid is an effective treatment though it does not always work.[5] In a case series study,[5] 4 patients (5 heels) with plantar fasciitis, who were unresponsive to palpation-guided injection, were treated with ultrasound-guided injection of triamcinolone acetonide. Four heels achieved complete relief. The results of our meta-analysis also demonstrated that patients treated by ultrasound-guided injection tended to suffer less pain and achieved higher response rate. Theoretically, under real-time visualization, ultrasound-guided injection provides more precise localization of the lesion and needle placement.[17] It is reasonable that ultrasound-guided injection achieves better outcomes.

Ultrasound provides objective measurements of efficacy on plantar fasciitis. The results showed that the decrease in the thickness and hypoechogenicity was more obvious in ultrasound-guided injection. The results further confirmed the efficacy of ultrasound-guided injection.

Two potential complications, fat pad atrophy and rupture of plantar fascia, occasionally occurred in plantar fasciitis patients treated with corticosteroids injection.[18] In all included studies, no atrophy was reported and significant difference was not detected with respect to heel pad thickness. The results were in accordance with Tsai,[17] who reported that heel pad thickness did not change after corticosteroid injection. It indicated that no heel pad atrophy occurred. The rupture rate of plantar fasciitis after corticosteroid injection ranges from 2.5%[19] to 6.7%[20]. However, no rupture was reported in all included studies. Thus, ultrasound-guided injection corticosteroid injection was an effective and relative safe method for patients with plantar fasciitis.

The study was based on the best evidence currently. However, some shortcomings should never be neglected. First, sample size was relative small. Theoretically, ultrasound-guided injection is more accurate and should get better prognosis. However, difference was not significant with respect to visual analogue score, heel tenderness index and response rate. We attributed it to the small sample size and the lack of evidence. Secondly, all included RCTs were not well designed. Although all studies mentioned randomization, most studies did not reported the randomize scheme, concealment of allocation and blinding methods. This might decrease the level of evidence. Besides, the power for VAS score was 0.7 approximately and the power for other outcomes were lower. It was a lack of evidence to prove the ultrasound-guided injection was superior to palpation-guided injection. Furthermore, heterogeneity was obvious with respect to response rate, heel tenderness index and heel pad thickness. It might be caused by different kinds of corticosteroids and no blinding methods for both therapists and patients. Finally, the outcomes varied with different practitioners. Namely, practitioners with more skills and experience might achieve better outcomes, especially for patients treated by palpation-guided injection. Despite there were some drawbacks, this study did reveal that patients treated with ultrasound-guided injection might suffer less pain and gain better results.

## Conclusion

Ultrasound-guided injection of corticosteroid tends to be superior to palpation-guided injection for the management of the plantar fasciitis. However, it is still a lack of evidence and more well-designed and large studies are needed to illustrate the issue.

## Supporting Information

Checklist S1 Completed PRISMA checklist.
(DOC)

File S1 Search strategy.
(DOC)

Table S1 Publication bias for all outcomes.
(DOC)
