# Supplementary material for: Ultrasound- versus Palpation-Guided Injection of Corticosteroid for Plantar Fasciitis: A Meta-Analysis
Source: PLoS One. 2014 Mar 21;9(3):e92671. doi: 10.1371/journal.pone.0092671 (PMC3962443; doi:10.1371/journal.pone.0092671)
Supplement: File S1 — Search strategy. (DOC) [file pone.0092671.s002.doc]

**File S1.** Search strategy.

The search strategy for Pubmed was as the following:

#1 "Fasciitis, Plantar"[Mesh]

#2 ((plantar fasciitis[Title/Abstract]) OR heel pain[Title/Abstract]) OR painful heel[Title/Abstract]

#3 #1 OR #2

#4 "Ultrasonography"[Mesh]

#5 ((ultrasound[Title/Abstract]) OR sonography[Title/Abstract]) OR ultrasonography[Title/Abstract]

#6 #4 OR #5

#7 "Palpation"[Mesh]

#8 (palpation[Title/Abstract]) OR unguided[Title/Abstract] OR blind[Title/Abstract]

#9 #7 OR #8

#10 #3 AND #6 AND #9
