# Supplementary material for: Ultrasound- versus Palpation-Guided Injection of Corticosteroid for Plantar Fasciitis: A Meta-Analysis
Source: PLoS One. 2014 Mar 21;9(3):e92671. doi: 10.1371/journal.pone.0092671 (PMC3962443; doi:10.1371/journal.pone.0092671)
Supplement: Table S1 — Publication bias for all outcomes. (DOC) [file pone.0092671.s003.doc]

**Table S1.** Publication bias for all outcomes.

| Outcomes | Publication bias (P value) |
| --- | --- |
| VAS | 0.58 |
| TT | - |
| HTI | - |
| Response rate | 0.73 |
| PFT | 0.11 |
| Hypoechogenicity | 0.37 |
| HPT | - |
